# Identification and Characterization of Highly Fluorescent Pigment Cells in Embryos of the Arabian Killifish (*Aphanius Dispar*)

**DOI:** 10.1016/j.isci.2020.101674

**Published:** 2020-10-13

**Authors:** Atyaf Hamied, Qusay Alnedawy, Ana Correia, Christian Hacker, Mark Ramsdale, Hisashi Hashimoto, Tetsuhiro Kudoh

**Affiliations:** 1 Biosciences, University of Exeter, Exeter, EX4 4QD, UK; 2 Department of Physiology, Development and Neuroscience, University of Cambridge, Cambridge, CB2 3EG, UK; 3 Division of Biological Science, Graduate School of Science, Nagoya University, Furo-cho, Chikusa-ku, Nagoya, 464-8602 Japan

**Keywords:** Specialized Functions of Cells, Embryology, Model Organism

## Abstract

The Arabian killifish, *Aphanius dispar*, is a small tropical teleost fish living in wide range of habitats in sea water and fresh water in the Middle East. Here, we report extraordinary fluorescent pigment cells in the Arabian killifish embryo. These cells appear brown in transmitted light, yellowish white in reflected light, and as strong fluorescence in GFP and RFP filters. TEM and confocal microscopy analyses show the fluorescence emanates from leucosome-like pigment organelles. The cells express the gene encoding GTP cyclohydrolase (*gch*), a marker for leucophores and xanthophore. Gene knockdown and knockout of *gch* using morpholino or CRISPR-Cas9 induced loss of fluorescence in these embryos, indicating a crucial role of the enzyme and the associated pterine biosynthesis pathway in the generation of the fluorescence. We concluded that these cells are a highly fluorescent subtype of leucophores and have named them as fluoroleucophores.

## Highlights

• Arabian killifish embryos possess extremely fluorescent pigment cells
• The fluorescent pigment cells show characteristics in common with leucophores
• Gene knockout of GTP cyclohydrolase removes fluorescence from the pigment cells
• The novel pigment cells are termed fluoroleucophores

## Graphical Abstract

**Figure:**
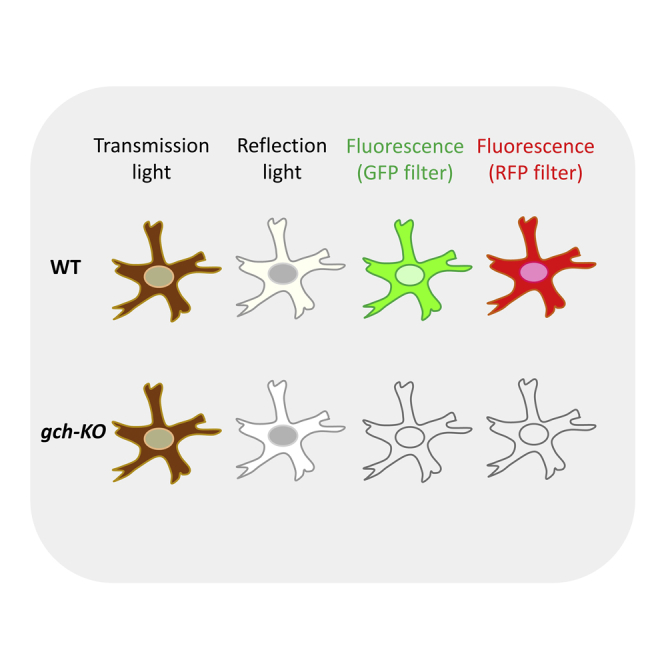

## Introduction

There are a variety of pigment cells in fish that create specific color patterns in each species and also protect the body from strong light (Armstrong et al., 2000; Kapp et al., 2018). Pigment cells in fish can be divided into two types as a result of their interaction with light. The first group absorbs light and includes melanophores, xanthophores, and erythrophores. The second group are cells containing light-reflecting pigments such as iridophores and leucophores (Fujii, 1993; Sugimoto, 2002). Melanophores contain pigmented organelles, melanosomes containing light-absorbing melanin pigment (Fujii, 1993). Leucophores and iridophores reflect light using their pigment organelles that contain crystalline purines and pteridines (Armstrong et al., 2000; Fujii, 1993; Hama, 1970; Kamei-Takeuchi and Hama, 1968). Although the difference is not always obvious, leucophores are generally considered to reflect light of all wavelengths producing a white color, whereas iridophores commonly create a broad wave length reflectors appearing silvery (Frohnhofer et al., 2013; Fujii, 1993). The differences between these pigment cells can be distinguished by examination of pigment organelles by TEM: Melanophores possess dark pigment granules with circular or oval shapes (Fujii, 1993). Leucophores contain leucosomes, circular granules containing dark spots (Menter et al., 1979; Takeuchi, 1976). Iridophores contain reflecting platelets, which appear as oblong structures in TEM sections with very pale contrast (Takeuchi, 1976). Xanthophores contain pterinosomes, which are also circular granules with relatively pale contrast (Fujii, 1993).

It has been known that during embryonic development, subsets of neural crest cells migrate from the periphery of the neutral plate/neural tube to locations all over the embryo and then differentiate into pigment cells (Donoghue et al., 2008; Kelsh et al., 2009; Le Douarin et al., 2004). In medaka (*Oryzias latipes*), there are shared characteristics between leucophores and xanthophores in relation to their developmental processes, both with respect to gene regulation and cell migration (Kimura et al., 2014; Nagao et al., 2014). The specific patterns of distribution of these different pigment cells contribute to the different and complex color patterns of the fish in embryos, larvae, and adults (Fujii, 1993; Kelsh et al., 2009). Recently, it has been reported that *Danio* species have two distinct leucophores, named xantholeucophores and melanoleucophores (Lewis et al., 2019). Xantholeucophores are orange pigmented cells, but melanoleucophores appear white, suggesting more complex variations of pigment cell types with potentially distinct and overlapping functions.

The Arabian killifish, *Aphanius dispar*, is a small teleost fish living in a wide range of coastal and stream habitats in the Middle East, variously establishing populations in sea water, brackish water, and fresh water (Haq and Yadav, 2011). The species has been used as a mosquito control agent (Al-Akel, 2011) and also as a model animal in toxicology studies (Saeed et al., 2015). Here we report on an extraordinarily fluorescent pigment cell found in the Arabian killifish embryo that we have named fluoroleucophores.

## Results

### Arabian Killifish Develop Highly Fluorescent Pigment Cells

**Figure 1:**
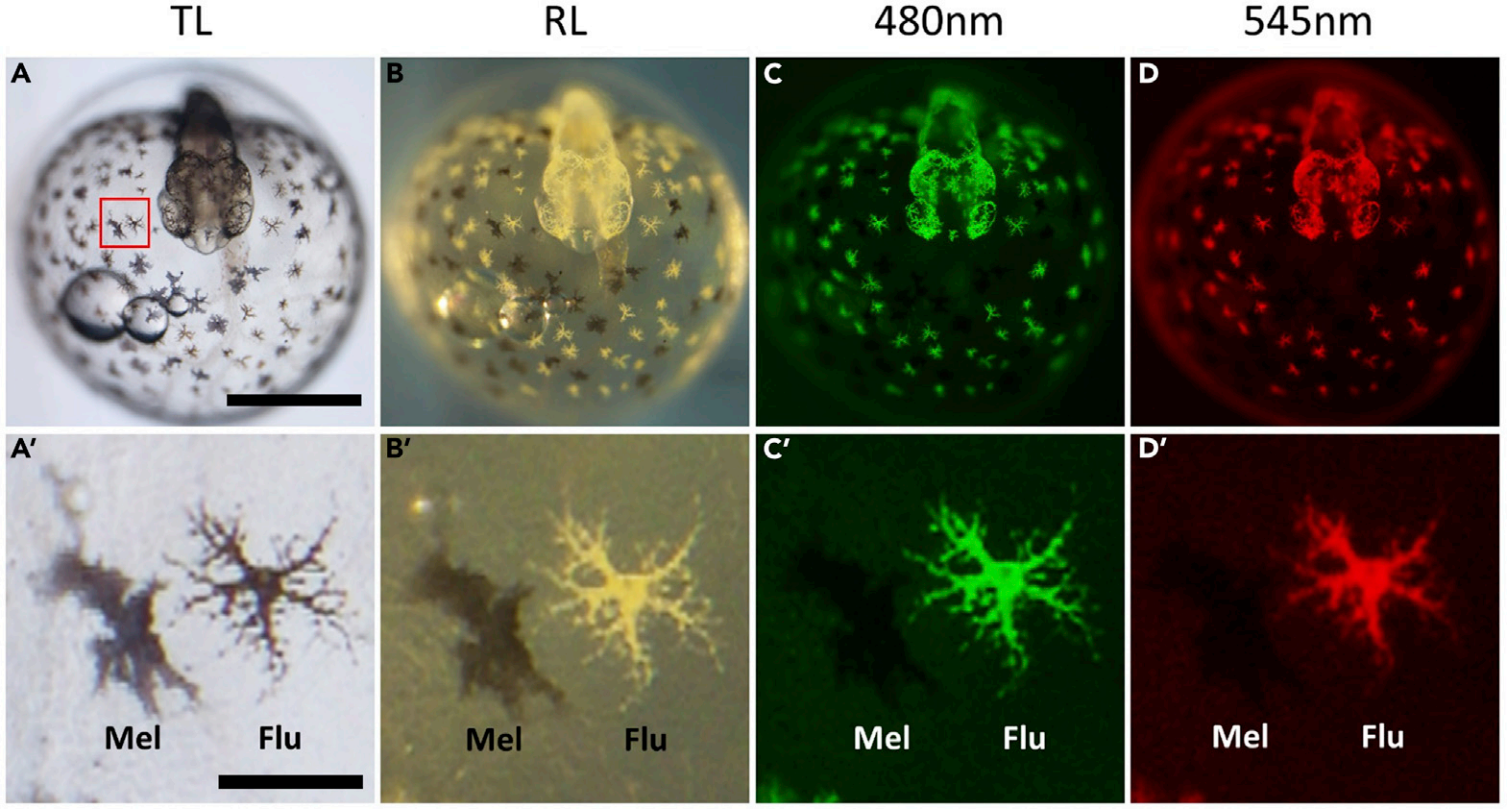
The Arabian Killifish Embryo Possesses Very Strongly Fluorescent Cells A 3-day post-fertilization embryo was imaged under transmitted light (A and A′), reflected light (B and B′), fluorescent light with GFP filter (C and C′), or RFP filter (D and D′). (A–D) Whole embryo image from the head dorsal view. (A′–D′) enlarged view of pigment cells melanophore (mel) and fluorescent cells (flu). Scale bars, 500 μm in (A–D) and 200 μm in (A′–D’).

**Figure 2:**
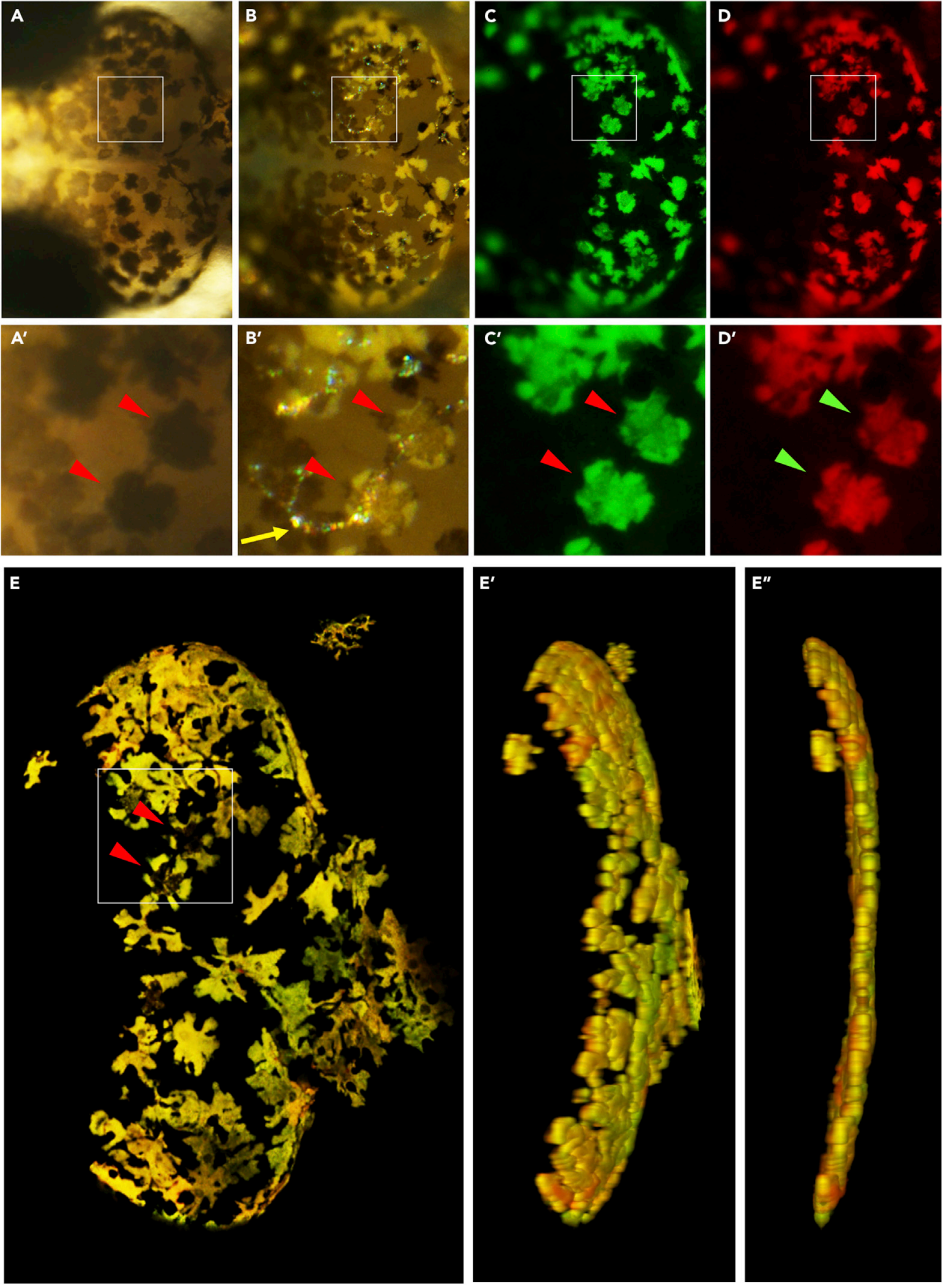
The Arabian Killifish Hatching Larva Possesses Very Strongly Fluorescent Cells Midbrain of a 12-day hatching larva was imaged using a stereo microscope (A–D) and confocal microscope (E). (A and A′) transmitted light, (B and B′) reflected light, (C and C′) fluorescent light with GFP filter and RFP filter (D and D′). (A–D) Whole midbrain, dorsal view with anterior left. (A′–D′) Enlarged view of pigment cells. Melanophore and fluorescent cells are highly overlapped (arrow heads). Iridophores are also partly overlapping with these cells (arrow). (E, E″, and E‴) The same larva was analyzed using confocal microscopy and 3D images were reconstructed from z stack. (E) is the original reconstruction, and (E′) and (E″) are rotated images of (E) showing there is only one fluorescent cell layer. The same fluorescent cells highlighted in stereo microscope (A′–D′) are also highlighted in the confocal image (E).

**Figure 3:**
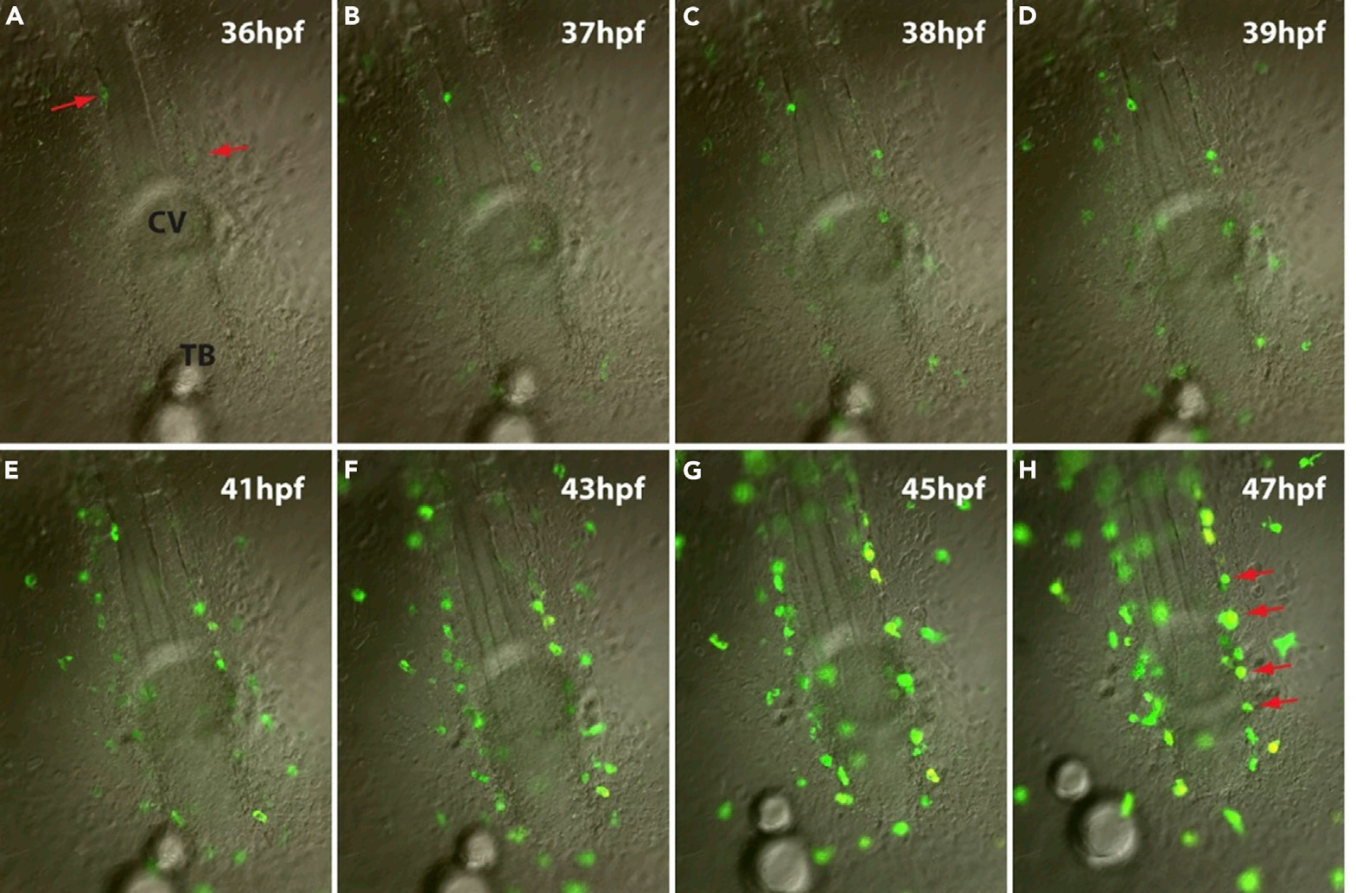
Fluorescent Cells Develop from the Early Somite Stage An early somite stage embryo with chorion was embedded in LMP agarose and imaged with time lapse (A–H). Image is focused on the posterior part of the embryo with the anterior upward. Fluorescent cells first appear at the border between the embryo and yolk (A, arrows) and migrate in the embryo and on the yolk. Newly developed pigment cells progressively appear more at the embryo border (H, arrow). Kv, Kupffer's vesicle.

Following observations of the development of Arabian killifish embryos, we noticed the presence of extremely fluorescent pigment cells that appear during somitogenesis stages (Figure 1). These cells look brown in transmitted light (Figure 1A) and yellowish white in reflected light (Figure 1B). However, under excitation with GFP and RFP fluorescent filters, the cells show a very strong fluorescence (Figures 1C and 1D). In post-hatched larvae, these pigment cells increase in number and spread all over the body. Figure 2 shows the mid-brain region of a post-hatched larva displaying a complex distribution pattern of pigment cells, with widely spread fluorescent cells. In addition, both melanophores with a black color and iridophores with iridescence overlap each other (Figure 2B’) suggesting that these three cell types may have a synergistic role to play. The same embryo was also imaged with a confocal microscope by which a 3D image was re-constructed from a z stack (Figure 2E). The reconstructions indicate that there is only single fluorescent cell layer located under the skin in the midbrain area. To trace the development of the fluorescent pigment cells, the earliest appearance of these cells was examined using time lapse imaging with differential interference contrast and fluorescence. Since Arabian killifish embryos and their chorions are both highly transparent, the embryos with chorions were embedded in low-melting-point agarose and imaged directly with time lapse (Figure 3). The fluorescent cells first appear soon at the onset of somitogenesis, which is far earlier than the initiation of pigment cell development reported in other model fish species including zebrafish, medaka, turquois killifish, and mangrove killifish (Api et al., 2018; Iwamatsu, 2004; Kimmel et al., 1995; Mourabit et al., 2011). These fluorescent cells first appear around the lateral and posterior edge of the embryo and subsequently appear on the surface of the yolk (Figure 3).

### The Fluorescent Pigment Cells Share Characteristics with Leucophores

**Figure 4:**
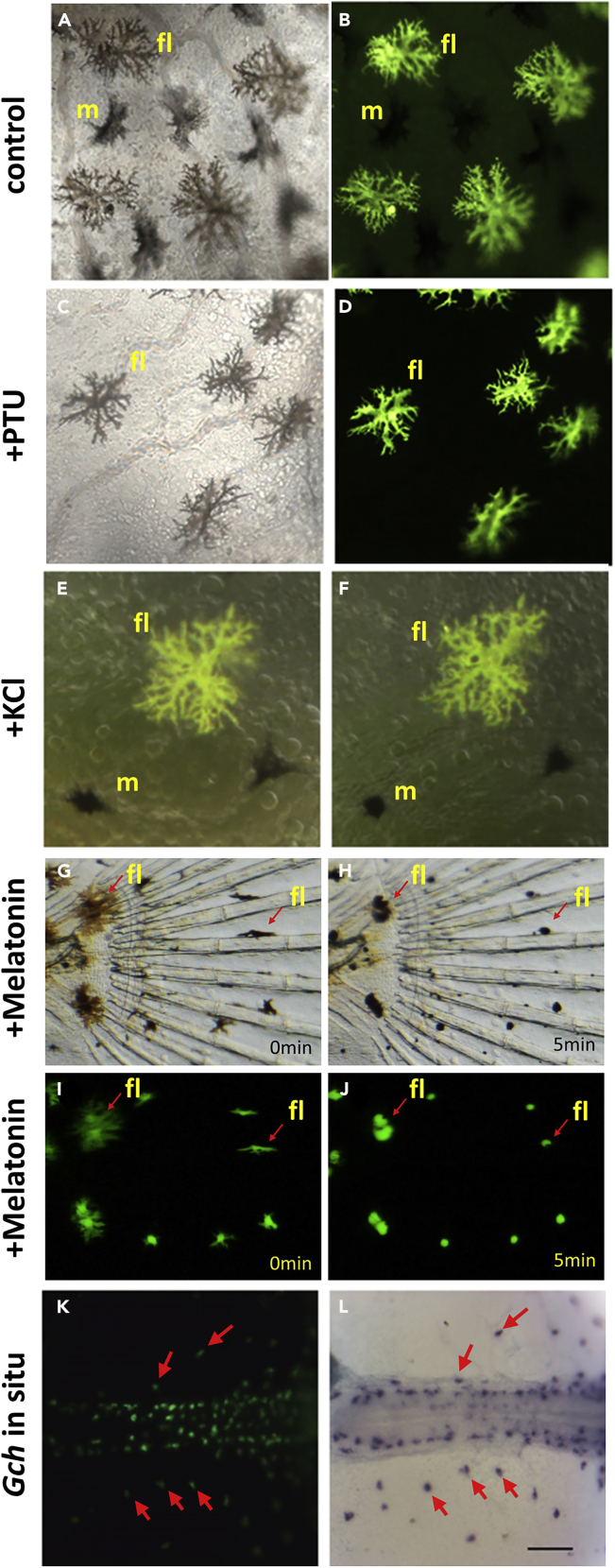
The Fluorescent Pigment Cells in *A*. *dispar* Share Common Characteristics to Leucophores Live embryo images in transmitted light (A, C, G, and H), fluorescent light with GFP filter (B, D, I, J, and K), and light of transmission light and fluorescence combined (E and F). (A, B) normal embryo having both black pigment cells (melanophore) and brown pigment cells (fluorescent pigment cells). (C and D) PTU-treated embryo lacks black pigmented cells but still has brown pigmented cells (fluorescent cells). (E and F) Before (E) and 5 min after (F) addition of 150 mM KCl. KCl induces aggregation of melanophores but fluorescent cells did not change. (G–J) Before (G and I) and after (H and J) addition of melatonin. Brown (fluorescent) pigment cells show aggregation by melatonin (I and J). (K and L) Live (K) and *in situ* stained (L) embryo with *gch* probe indicating fluorescent cells express *gch*. Scale bar is 100 μm in (K) and (L).

To distinguish the behavior of the black and brown (fluorescent) pigment cells, melanin synthesis was blocked using 0.003% phenylthiourea (PTU) (Figures 4C and 4D) and compared with the control embryos (Figures 4A and 4B). The data show that cells with black pigment were not seen in the PTU-treated embryos, but the brown pigment cells were still there, suggesting that the brown pigment cells do not contain melanin. When embryos were exposed to potassium ions, the black pigments become aggregated as previously reported in melanophores in other species (Fujii, 1993) but the brown pigment did not (Figures 4E and 4F), confirming that only the black pigment cells have the characteristics of melanophores. To further distinguish between these two pigment cells, embryos were treated with melatonin, causing compression of the brown/fluorescent pigment cells (Figures 4G–4J). Overall, the responses of the brown pigment cells are similar to those of leucophores from other species (Fujii, 1993). To further test if the fluorescent cells are related to leucophores, gene expression of GTP cyclohydrolase (*gch*), a marker of leucophores and xanthophore (Nagao et al., 2014), was examined using *in situ* hybridization at an early somite stage. Data show that fluorescent pigment cells do express *gch* (Figures 4K and 4L). As these cells have similar characteristics to leucophores in shape, color pattern, contractive behavior, and gene expression but have increased fluorescence, we have named these cells as fluoroleucophores.

### The Fluorescence Originates from Leucosomes within the Fluoroleucophores

**Figure 5:**
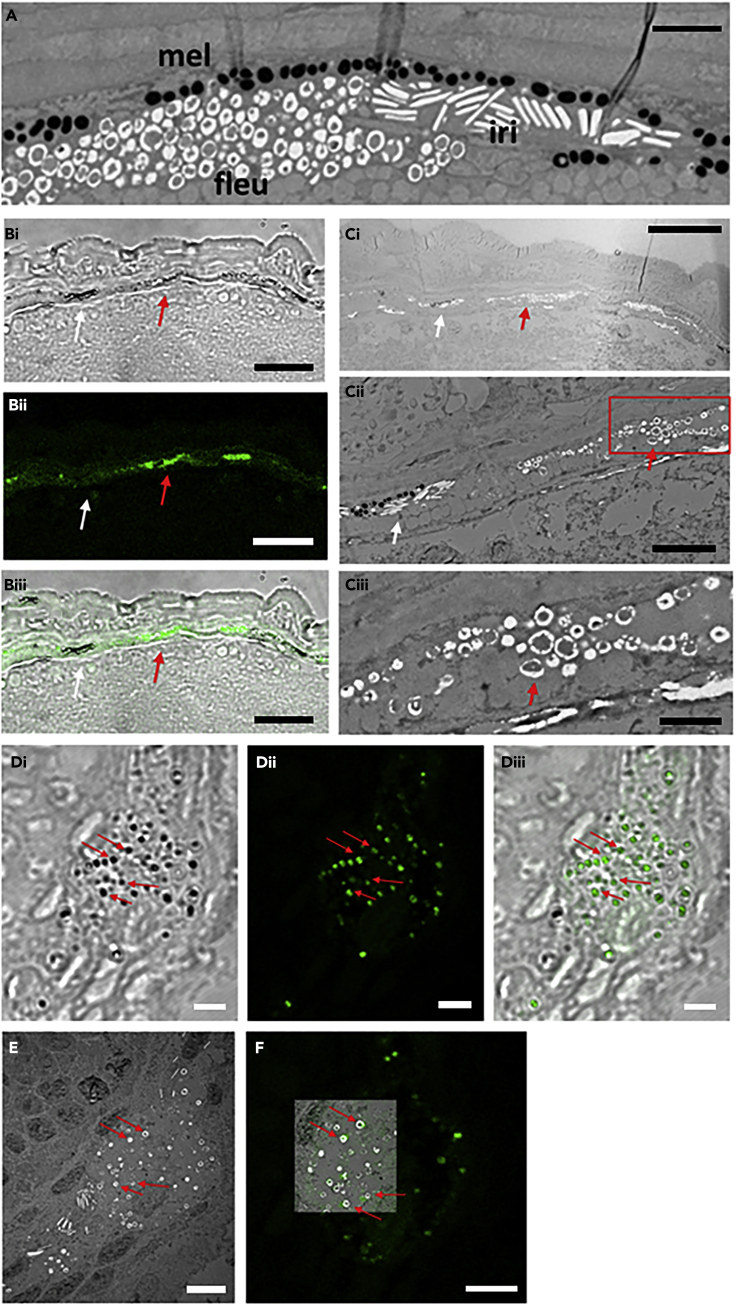
The Fluorescence Comes from a Pigment Granule, the Fluoroleucosome in the Fluoroleucophore Hatching larvae (12 dpf) were fixed with 2% glutaraldehyde and 2% paraformaldehyde and analyzed by TEM. (A) Distinct pigment cells; melanophores (mel) with black granules, melanosomes, iridophores (iri) with reflecting platelets, and fluoroleucophores (fleu) with fluoroleucosomes. (B–E) Hatching larva fixed with 4% paraformaldehyde and embedded in LR-white. Adjacent sections were analyzed in confocal microscopy (B, D), TEM (C, E), and overlay of confocal and TEM images (Dii and E, respectively) in (F) showing overlapping of fluorescence with fluoroleucosomes (red arrow) but not with reflecting platelets in the iridophores (white arrow). Scale bars, 2 μm (A, Ciii, D, E), 20 μm (B), 50 μm (Ci), 5 μm (Cii).

To identify the source of fluorescence at subcellular levels, 12 dpf larvae were fixed with 2% glutaraldehyde and 2% paraformaldehyde and analyzed using TEM (Figure 5A). Images of the surface of the midbrain show cells having different pigment organelles: melanosomes (black organelles) in the melanophore, reflecting platelets (white and long organelles) in the iridophore, as well as leucosome-like pigment granules (round white organelles with a dark spot). To examine if these cells do correlate with the observed fluorescence, 12 dpf larvae were also lightly fixed with 4% paraformaldehyde and embedded with LR White plastic resin, which can preserve fluorescence (Bell et al., 2013). Two adjacent sections were analyzed using a combination of confocal microscopy (Figures 5B and 5D) and TEM (Figures 5C and 5E) allowing direct comparison of fluorescent domains and ultrastructural composition of those areas. Images of the surface of the midbrain region reveal that the fluorescent domain coincides and overlaps with domains of leucosome-like granules but not with iridophore reflecting platelets; suggesting that fluorescent cells are leucophores (fluoroleucophores) but not iridophores (Figures 5B and 5C). With an enlarged view, individual granules are seen in the DIC as dark dots (Figures 5Di) and with fluorescence under confocal microscopy (Figures 5Dii). TEM images from the adjacent ultrathin sections show that these fluorescent dots overlap with the position of individual leucosome-like granules (Figures 5E and 5F). These data indicate that the fluorescence indeed comes from these leucosome-like granules.

### Fluorescence in the Arabian Killifish Leucophore Is Approximately Five Times Higher Compared with that Seen in Medaka

**Figure 6:**
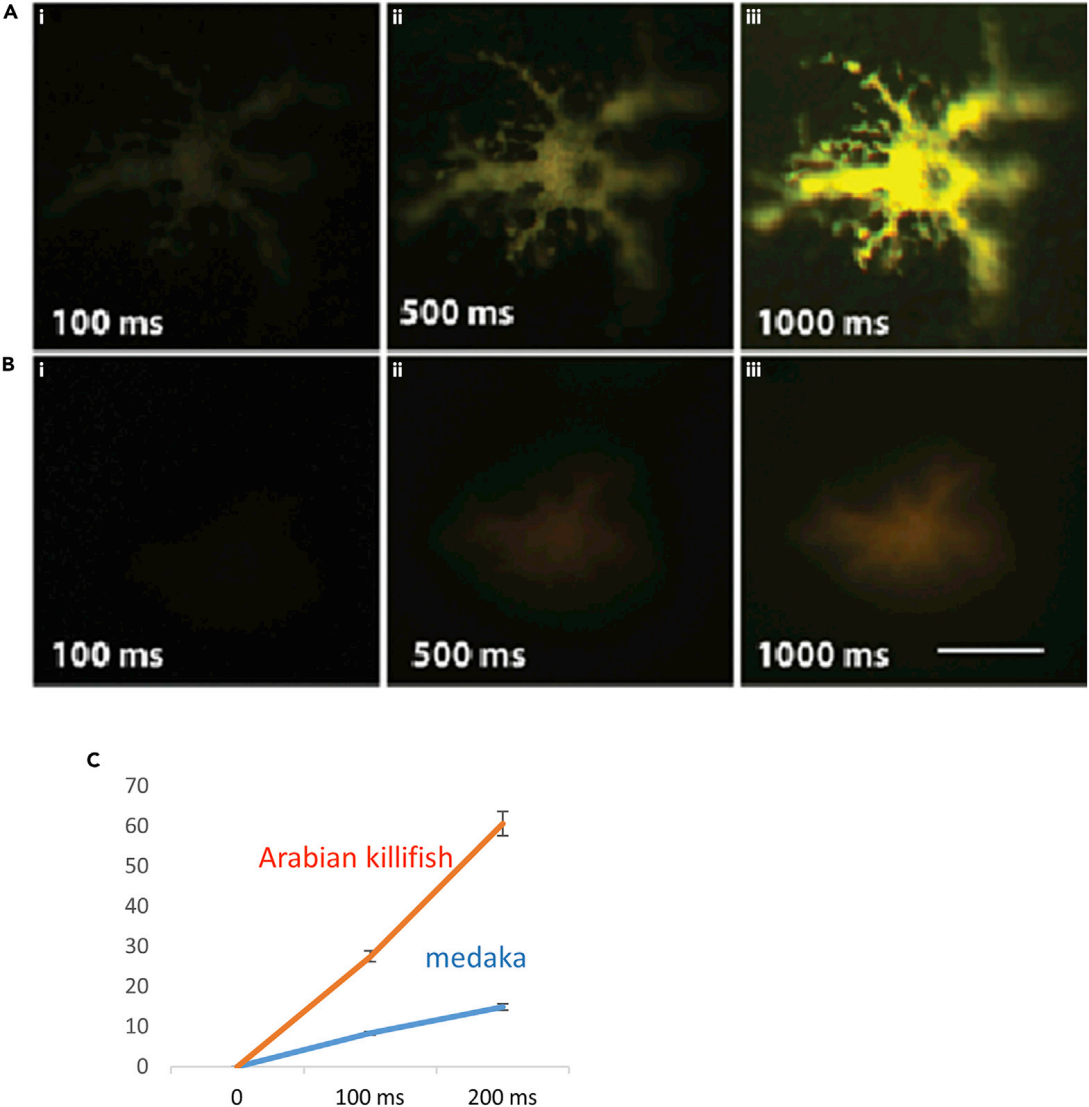
The Leucophore in *A. dispar* Is Far Brighter than in Medaka The fluorescent pigmentation with the Arabian killifish fluoroleucophore (A) and medaka leucophore (B) were imaged with different acquisition times (20–1,000 ms). (C) Each point is the mean of three biological replicates ±SE. Linear curve of fluorescence and acquisition time show the Arabian killifish fluoroleucophores are approximately 5-fold brighter than the medaka leucophores. Scale bar is 100 μm.

To quantitatively compare the fluorescence intensity of fluoroleucophores in the Arabian killifish with leucophores from other species, images of fluorescent pigment cells were taken from the embryos of the Arabian killifish and medaka with fixed acquisition times; light intensity was then quantified using ImageJ (Figure 6). With short acquisition times (100 or 200 ms), light intensity was consistently higher in the Arabian killifish leucophore by approximately 5-fold (Figure 6C). At longer acquisition times, relative light intensity decreased owing to saturation of the image (Figure 6Aii and 6Aiii). These data indicate that the fluorescence of the fluoroleucophore in Arabian killifish is much higher than the leucophore in medaka.

### GTP Cyclohydrolase Is Essential for Synthesis of the Fluorescent Pigment in the Arabian Killifish Fluoroleucophore

**Figure 7:**
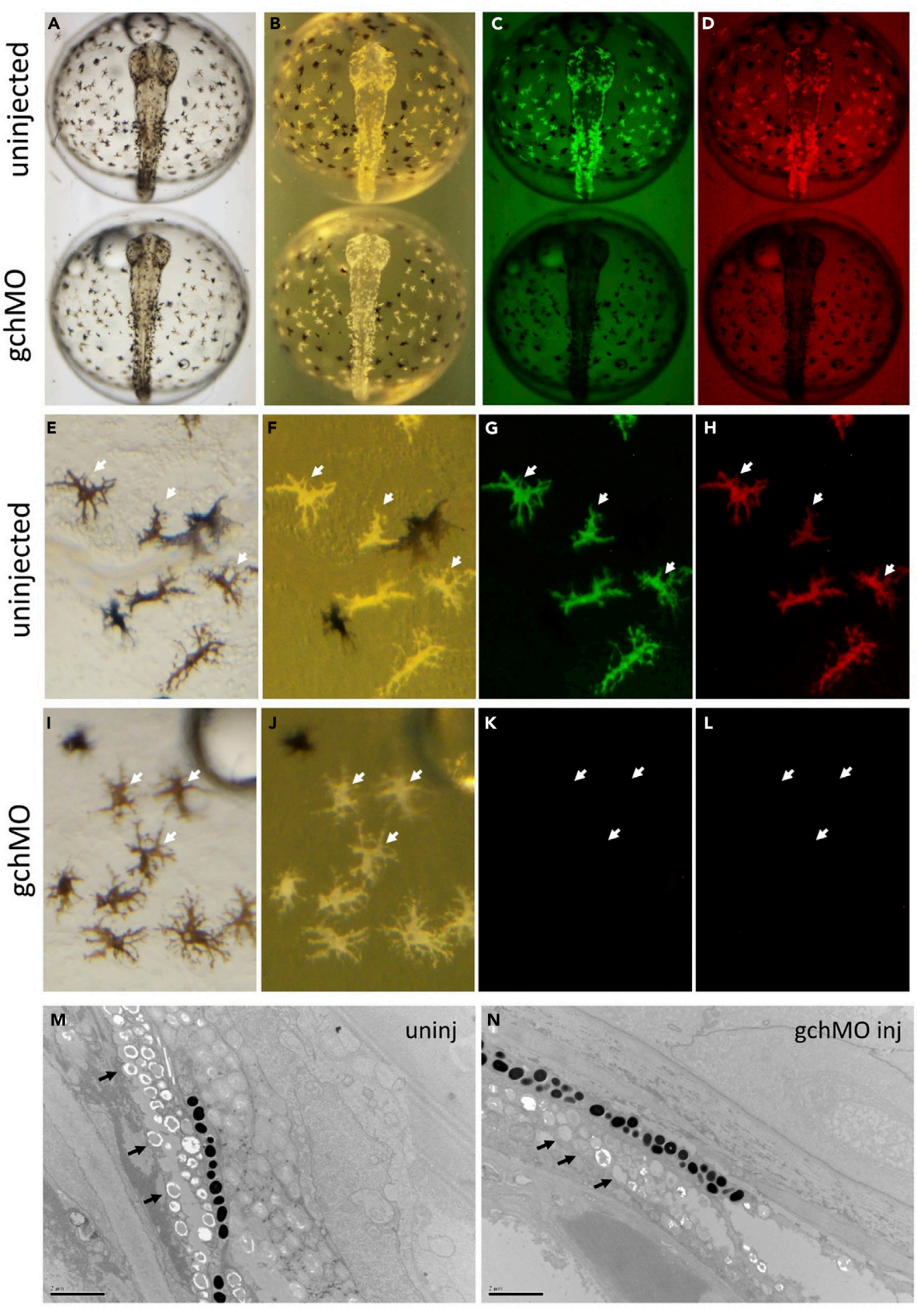
*Gch* Morpholino Blocks Development of Fluorescence in the Fluoroleucophore Live embryos at 3 dpf imaged with transmitted light (A, E, I), reflected light (B, F, and J) or fluorescent light with GFP filter (C, G, and K) and RFP filter (D, H, L). (A–D) Upper embryo (control) shows fluorescence (C and D) but lower embryo (*gch*MO) shows no fluorescence. (E–L) Enlarged images show fluoroleucophore is yellowish white (F) and fluorescent (G and H) in the uninjected control, but pale white (J) and non-fluorescent (K and L) in *gch*MO-injected embryos. (M and N) Control and *gch*MO larvae at 12 dpf were analyzed with TEM. In normal embryos, fluoroleucosomes show a pale background with a dark spot (M, arrow) but such structures are mostly lost in the *gch*MO and instead lightly stained granules become major granules in the fluoroleucophore (N, arrow). Scale bars, 2 μm (M, N)

It has been reported that knock out of a GTP cyclohydrolase causes a loss of fluorescent pigmentation in the xanthophores of zebrafish (Lister, 2019). Although the level of fluorescence in xanthophores is much lower, and the morphological characters of the pigment granule are different, it was hypothesized that *gch* may have a role in generating fluorescent pigments in the fluoroleucophore as well. To test this possibility, *gch* was knocked down by injecting a *gch* specific morpholino antisense oligonucleotide. In these embryos, the fluoroleucophores are still visible in transmission light with a brown color (Figures 7A and 7I) and in the reflected light with a whitish color (Figures 7B and 7J). However these cells, which are normally fluorescent (Figures 7C, 7D, 7G, and 7H), have all lost fluorescence (Figures 7K and 7L) following knockdown of *gch*. In addition, the whitish yellow color seen in the controls under reflected light (Figure 7F) is altered to a paler white color (Figure 7G). To examine if the morphology of the fluoroleucosome is affected by the knockdown of *gch*, the organelle was observed by TEM. Indeed, the fluoroleucosomes that have a pale background with a dark spot inside were highly reduced and instead granules with different morphology (greyish colored, no dark spot inside) became the major granules in the cell. Therefore, the observed reduction in number of normal fluoroleucophores seems consistent with the reduction of fluorescence.

**Figure 8:**
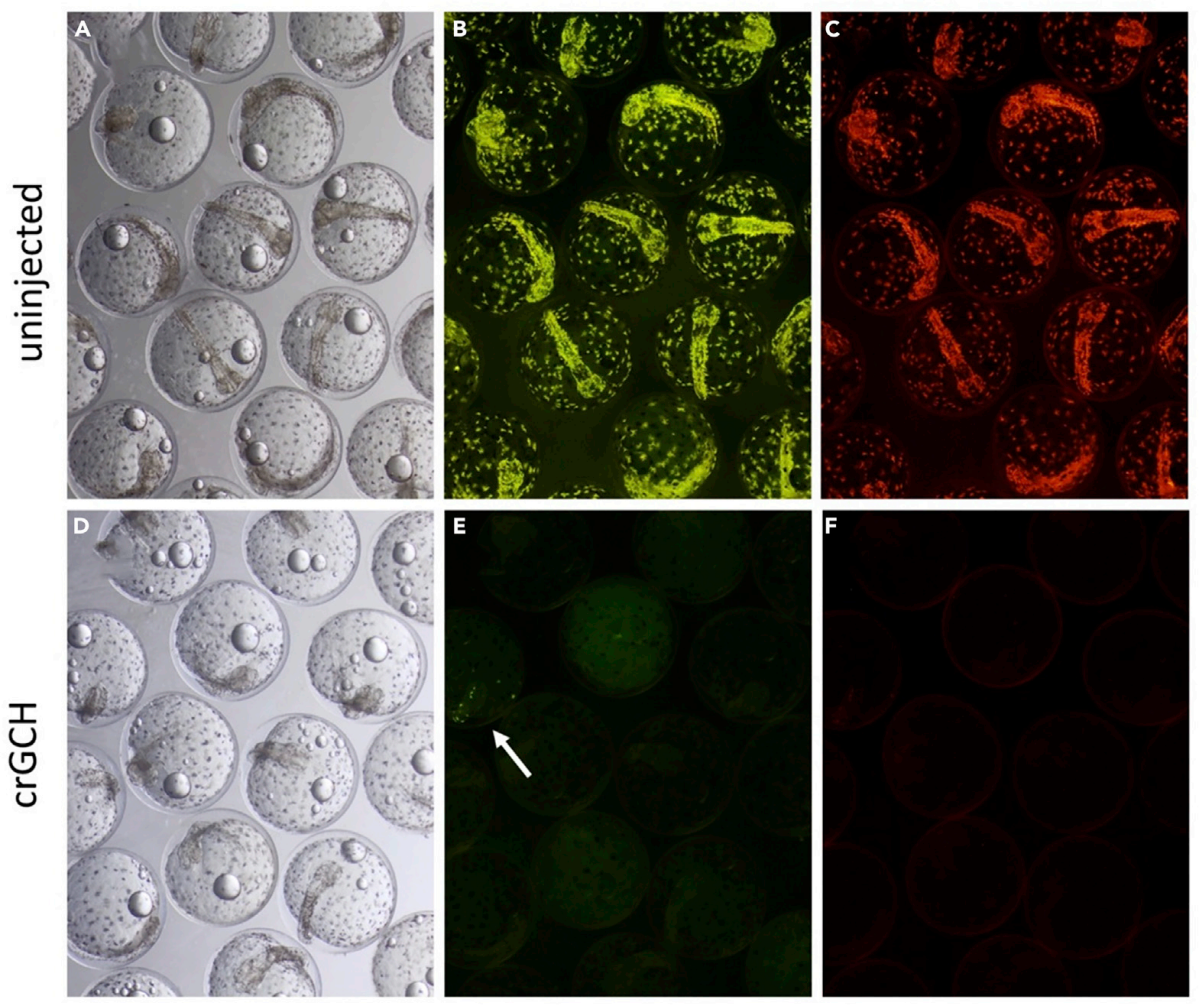
*Gch* Gene Knockout by CRISPR-Cas9 Induces Loss of Fluorescence Embryos (3 dpf) imaged with transmitted light (A and D), fluorescent light with GFP filter (B and E), and RFP filter (C and F). Control uninjected embryos show fluorescence (A–C) but all of *gch* crRNA injected embryos show reduced fluorescence (D–F). Arrow indicates a small number of fluoroleucophores that are still faintly fluorescent in the crRNA injected embryos.

To further confirm the crucial role of *gch* in the generation of the fluorescence, the *gch* gene was also knocked out by injecting CRISPR RNAs (Figure 8). Consistent with the morpholino data, all injected embryos lost fluorescence (Figures 8E and 8F).

## Discussion

### Characterization of the Fluoroleucophore in the Arabian Killifish

Here we showed that Arabian killifish embryos possess highly fluorescent pigment cells. The cells are brown in transmitted light, yellowish white under reflected light, and strongly fluorescent when examined with GFP and RFP filters. The characteristics of the cells and the specific gene expression patterns for *gch* (Kimura et al., 2014) suggest that these cells are related to leucophores. Owing to the extremely high fluorescence observed, we named the cells as fluoroleucophores. These cells might be an evolutionary adaptation of the leucophore in this species to allow the fish to live in environments with strong sun light. It should also be noted that the fluorescent pigment cells develop at very early stage at the onset of somitogenesis that is much earlier than the first appearance of leucophores in medaka at stage 25, 50 hpf (Lynn Lamoreux et al., 2005). We have also examined the embryonic development of pigment cells in other fish species including zebrafish, mangrove killifish, turquois killifish, bluntnose knifefish, clownfish, and rainbow trout (Alshami et al., 2020; Api et al., 2018; Finch et al., 2010; Ghosh et al., 2009; Mourabit et al., 2011). In these fish species, visible pigmentation under transmission or reflection light only occurs at the late somite stage or later. This also suggests that intense pigmentation from the earliest stage of embryonic development in this species may be a specific adaptation necessary for survival in harsh environments. It has been reported that xanthophore pigmentation is enhanced by UV exposure (Le Guyader and Jesuthasan, 2002). It is possible that the Arabian killifish may have developed stronger reflecting pigment cells to resist to strong light condition in their habitat in the Middle East. It may also be possible that the fluorescent pigment is used as a visual signal, for instance, for mating behavior.

Potentially there could be some overlap of function and ontogeny between leucophores and xanthophores. Both lineages come from common progenitor cells (Kimura et al., 2014; Nagao et al., 2014). In *Danio* species, a sub-type of the leucophore (xantholeucophore) contains pteridines and carotenoids (Lewis et al., 2019) like xanthophores. Both cell types express *gch*, supporting the notion that fluoroleucophores may have overlapping characters and roles with xanthophores. The evidence from different teleost fish species suggests that pigment cells have many different variations, which have some diverse but also have overlapping functions. It is therefore conceivable that each species living in different light environments may use a variety of pigment cells to cope with the conditions they find themselves in.

Although highly fluorescent cells have not been widely described in fish species, fluorescence was observed in several adult fish species including cat shark, gobies, and moray eels (Gruber et al., 2016; Sparks et al., 2014). However, the description of fluorescence in these fish is at the organismal level but not at the cellular level, and therefore fluorescent cells have not been characterized in detail before. In addition, comparing the fluorescent level between the skin in a large adult fish and individual embryonic pigment cell is not straight forward. Further research is therefore needed to identify fluorescent cells in a variety of fish species, identify their common and diverse characters, and to quantitatively compare the fluorescent levels.

In medaka it has been revealed that K^+^ induces aggregation of pigment granules in the melanophore and xanthophore and dispersion in the leucophore (Iga, 1978). However, in the Arabian killifish, K^+^ induced aggregation of the melanophore but did not induce any change (aggregation nor dispersion) in the fluoroleucophore. This is another difference of the fluoroleucophore in Arabian killifish to leucophore known from other species. This may suggest complicated diversity of pigment cell development and evolution in different teleost fish species.

*Gch* is a well-known marker for leucophores and xanthophores (Nagao et al., 2014), with the GTP cyclohydrolase acting as a key enzyme for biosynthesis of biopterin. In zebrafish, it has been reported that mutation in *gch2* causes a deficiency in the pigmentation of the xanthophore (Lister, 2019). These data suggest an important role for GTP cyclohydrolase in generating pterine-based fluorescent pigments. It may also suggest that leucophore and xanthophore are related in cell lineage as previously discussed from gene expression, signaling, and morphology (Fukamachi et al., 2006; Kimura et al., 2014; Nagao et al., 2014). However, fluorescence in xanthophores is very weak, whereas the fluorescence in leucophores is much stronger, with the fluorescence in the fluoroleucophore of the Arabian killifish being five times stronger than medaka. It is still not clear if such a difference in strength in fluorescence is due to the different pigment molecules or the quantity of a shared molecule. The fact that white reflection still occurs in the fluoroleucophore in *gch* morphants and crispants may suggest that white reflecting pigment(s) and the fluorescent pigment(s) are different molecules and only the fluorescent molecules (possibly pterine [Armstrong et al., 2000; Lewis et al., 2019; Oliphant and Hudon, 1993]) are synthesized via the GCH pathway. In that case, the white pigment molecule (which might be uric acid [Hama, 1975]) would still be present in the *gch* morphants and crispants.

In the initial stages of development of fluoroleucophores and melanophores at somitogenesis stages, the newly developed pigment cells are physically separated from each other, with spaces between the same cell types as well as between the two cell types (image in Figures 1A’and 1D′). On the other hand, at the stage of hatching (12 dpf) fluoroleucophores and melanophores seem to specifically overlap (Figures 2A’ and 2D′). At this stage, separation and spacing between two next fluoroleucophores or between two melanophores are still maintained (Figures 2A–2D). This suggests that there is both a homo and a hetero repellent mechanism between pigment cells at early embryonic stages, whereas at the hatching stage the homo repellent mechanism is still prominent but the hetero repellent mechanism is lost between fluoroleucophores and melanophores. Indeed, not only is the repellent mechanism lost, but also more specific hetero attraction seems to occur (Figures 2A–2D). At early stages when these pigment cells develop from small areas of the neural crest and when these cells need to be quickly distributed all over the embryo, homo and hetero chemo repellent mechanisms would be useful. Once these cells are distributed, these different pigment cells may need to function as a unit, for instance, to deal with strong UV light from early stage of development (Armstrong et al., 2000; Kapp et al., 2018); therefore, hetero attraction may initiate (Figure 2B’). Tight association between leucophore and melanophore is also reported in other species such as medaka (Lynn Lamoreux et al., 2005).

### Arabian Killifish Embryos as a Model for Embryological Imaging of Pigment Cells

Arabian killifish embryos are highly transparent, and although they have similar transparency to the medaka embryo, the chorion of Arabian killifish is smooth and transparent and does not have the hairs that medaka chorion has facilitating imaging. The number of oil droplets is also less than in medaka embryos. In addition, the embryos at the somite stages and earlier are highly stable because they do not share the rhythmic contraction of the embryos seen in Medaka. Overall, therefore, higher quality embryonic imaging with both transmission light and fluorescent light can be achieved at the single cellular level without removing the chorion (e.g., Figures 1, 4, and 7). In other popular model fish species, development of pigment cells occurs at a later stage, so in the Arabian killifish, fluorescent cells can be tracked with optical clarity, without anesthesia and without using any labeling techniques from an early somite stage, mainly from a domain around the neural crest to their terminal destination. The Arabian killifish is therefore an excellent model for studying pigment cell development and function in fish.

### Limitations of the Study

Our data indicate crucial role of the enzyme, Gch, and suggest the fluorescent pigment in the Arabian killifish being a type of pteridine. However, the exact nature of the molecule is yet to be identified. Biological roles of the fluoroleucophore are also to be investigated in future studies.

### Resource Availability

#### Lead Contact

Further information and requests for resources and reagents should be directed to and will be fulfilled by the lead contact, Tetsuhiro Kudoh: t.kudoh@exeter.ac.uk.

#### Materials Availability

No unique reagents have been generated in this study and all the materials are commercially available.

#### Data and Code Availability

The data that support the findings of this study are available from the Lead Contact on reasonable request.

## Methods

All methods can be found in the accompanying Transparent Methods supplemental file.

## Supplemental Information

Document S1. Transparent Methods
