# Supplementary material for: Identification and Characterization of Highly Fluorescent Pigment Cells in Embryos of the Arabian Killifish (Aphanius Dispar)
Source: iScience. 2020 Oct 13;23(11):101674. doi: 10.1016/j.isci.2020.101674 (PMC7593555; doi:10.1016/j.isci.2020.101674)
Supplement: Document S1. Transparent Methods [file mmc1.pdf]

iScience, Volume *23*

Supplemental Information

Identification and Characterization of Highly Fluorescent Pigment Cells in Embryos of the Arabian Killifish (*Aphanius Dispar*)

Atyaf Hamied, Qusay Alnedawy, Ana Correia, Christian Hacker, Mark Ramsdale, Hisashi Hashimoto, and Tetsuhiro Kudoh

Materials and Methods

Arabian killifish (*Aphanius dispar*)

Arabian killifish were kept in a recirculation system (Artificial Sea Water/ASW 35 ppt at 26.0°C) in the Aquatic Resource Centre at the University of Exeter. To collect eggs, a glass chamber was placed in the fish tanks in the morning. Eggs were collected from the chamber after an hour and transferred to Petri dishes with 20 ml of Artificial Sea Water (ASW) and were incubated at 26°C with daily water changes. For live imaging, embryos were kept submerged in “channel gels” made by leaving agarose to set on 1.2 mm diameter glass tubes [19]. All photographs were taken using an Olympus, SZX2-ILLK microscopes.

Medaka (*Oryzias latipes*)

Medaka were maintained in RO water with a recirculation system. The fish spawn eggs in the morning. Eggs were manually removed from the fish caught in a fish net. Hairs on the chorion were removed with tweezers. Eggs were incubated with RO water at 26°C.

In situ hybridisation

Embryos with chorions intact were fixed with 4% *para*-formaldehyde (PFA) in ASW at 4°C for 4 days. Chorions were manually removed using tweezers in phosphate buffered saline (PBS), and stored in methanol at -20°C. The Medaka *gch* probe [14] was digested with NotI and transcribed with Sp6 RNA polymerase. *In situ* hybridisation of Arabian killifish embryos was conducted using a protocol for the Mangrove Killifish, *Kryptolebias marmoratus* [19].

Transmission Electron Microscopy

For ultrastructural analysis, embryos were fixed in 2% glutaraldehyde and 2% PFA in 0.1 M sodium cacodylate buffer (pH 7.2) and stored at 4°C until further processing. Embryos were then washed 3 x 5 min in buffer, post-fixed in 1% osmium tetroxide (reduced with 1.5% potassium ferrocyanide) in buffer then washed again for 3 x 5 min in deionized water. Embryos were then dehydrated in a graded ethanol series and embedded in Spurr resin. Ultrathin sections (60 nm) were obtained using an ultramicrotome (EM UC7, Leica) and then placed on pioloform-coated copper slot grids (Agar Scientific) and contrasted in lead citrate before analysis on a JEOL JEM 1400 TEM. Images were taken with a digital camera (ES1000W, Gatan, Ametek).

To correlate ultrastructure with fluorescence signals, embryos were embedded using a protocol established by Bell *et al*. (2013). In brief, embryos were fixed in 4% PFA in 0.05 M PIPES buffer (pH 7.2) at 4°C in the dark. All subsequent steps were also completed in the dark at 4°C. After 3 x 10 min washes in buffer (including 1 mM $CaCl_2$), embryos were dehydrated in an ethanol gradient containing 1 mM DTT until 90% ethanol was attained; embryos were then gradually embedded in LR white resin. At the final resin incubation step, samples were brought to room temperature and samples polymerised for 24 h at 50 degrees. For correlative light and electron microscopy, 500 nm thick sections were collected on glass slides and were imaged with a confocal microscope (Zeiss LSM 880). Directly adjacent sections were taken at 60 nm, contrasted with lead citrate and could be imaged with the TEM. Images were superimposed based on structural landmarks of the bright field image and TEM micrographs using Photoshop.

Microinjection of gch morpholino or *gch* crRNAs

A morpholino for *A. dispar gch* (*gch*_MO) that targets the start codon region was purchased from Gene-Tools (TTCATGTCGGCTTTGGCTTGTTTG). The morpholino was injected at 2 µg/µl, with an injection volume of approximately 1 nl using a WPI microinjector. CrRNAs (Alt-R CRISPR-Cas9 crRNA, IDT) for *A. dispar gch* were designed to target two domains in the protein coding regions (*gch*_crRNA1 GTCCCGCTTACCCGCTCTGG and *gch*_crRNA2 GAGGAACTGAATGGCCTTGG). A mixture of two crRNAs, at 50 ng/µl each, 100 ng/µl tracrRNA and 200 nM Cas9 nuclease (NEB), were injected into the 1-cell stage embryos.

Ethics Statement

All experiments were approved and performed in compliance with the regulations of the University of Exeter, Animal Welfare Ethical Review Board.
